# Endoscopic treatment of vesicoureteral reflux in a paediatric surgery ambulatory unit

**DOI:** 10.4103/0972-9941.78344

**Published:** 2011

**Authors:** Fernando Rivilla

**Affiliations:** Division of Paediatric and Urology Surgery, San Carlos University Hospital, Madrid, Spain

**Keywords:** Endoscopic treatment, paediatric ambulatory surgery, paediatric day surgery, vesicoureteral reflux

## Abstract

**BACKGROUND:**

Vesicoureteral reflux (VUR) is a major urological problem in children. Its incidence ranges from 1 to 3% in healthy children.

**MATERIALS AND METHODS:**

We treated 38 children and analysed their data on age, sex, reflux grade, laterality, and results of endoscopic treatment (ET), at the different grades of reflux. All children were operated on an Ambulatory Surgery basis, studying the complications and post-operative course.

**RESULTS:**

Thirty-eight patients were operated during a period of six years, of age between one and twelve years. VUR was bilateral in 24 (63%) patients, unilateral in 14 (34%), with a collection of a total of 62 renal units or ureters. In 29 children (76%), 46 refluxing ureters (70%) completely disappeared after just 1 ET. Nine patients (24%) with 16 ureteral units (30%) received a second ET, with the reflux disappearing successfully in seven children (12 ureteral units), changing the success rate in the disappearance of VUR, after two injections of Deflux, to 90% of the total group of ureters (58 of 62).

**CONCLUSION:**

The endoscopic treatment of VUR has become the first choice of treatment to control the primary reflux, not just because of the good results, but because of the low post-operative morbidity and the direct relationship with the Ambulatory Surgery Unit.

## INTRODUCTION

Vesicoureteral reflux (VUR) is a major urological problem. The incidence ranges from 1 to 3% in healthy children and between 30 and 50% in children who have had urinary tract infection (UTI). If not properly treated, recurrent UTI can cause scarring of the kidneys, which could evolve into chronic renal failure.[1]

Traditionally, medical treatment was through the use of prolonged urinary chemoprophylaxis, to prevent the recurrence of UTIs and thereby avoid renal damage and promote child growth and maturation of the physiological mechanism. Surgical treatment is indicated, inter alia, where the patient has recurrent UTI or there is a renal damage. This treatment is through different open techniques designed to restore the physiological antireflux mechanism, which would rebuild an adequate submucosal tunnel length of the intramural ureter, at the trigone.[1]

The concept of ET of VUR, by injecting a sub-ureteral substance favouring the physiological antireflux mechanism is not new, since it was first described in 1981, by Matouschek[2] and later popularised by O’Donnell and Puri in 1984, by publishing a study on a large series of patients.[3] Since then, numerous clinical experiences have been published worldwide, with significant results, which have altered the strategy of surgical treatment of reflux, with a prompting to choose open surgery.

For a similar or higher efficiency compared to open surgery, ET has been introduced today as the choice treatment for VUR in most cases.[4]

There are several reasons for choosing ET, such as the technical simplicity of its application, the smaller number and importance of post-operative complications, the greater acceptance of the patient and family and the possibility for it to be performed at a Unit for Paediatric Ambulatory Surgery.

The substances that have been used for implementation length sub-ureteral have varied over recent years in order to improve their molecular characteristics, so that was not enough antigenic over time, unable to migrate from the place of its application and it was easily mastered by a surgeon. Teflon injection was the first material that was used, and thereafter, others such as Macroplastique (Dimetilpolixilosano), chondrocytes and bovine collagen were used, however, Deflux (dextranomer Hyaluronic Acid), appears to be the most used today, as it brings together most of the above-mentioned properties.[5]

Outpatient surgery has grown exponentially in recent years to include in its portfolio of services many more different types of lesions, which are surgically treatable on an outpatient basis. Therefore, surgical treatment of VUR has been one more of those that have adopted its location in a Paediatric Day Hospital or a Unit of Paediatric Ambulatory Surgery, both in the open surgical treatment modality using minimally invasive techniques or by an ET.[6,7]

The aim of this article is to describe our experience from the Ambulatory Unit in the ET of children with VUR through the various stages of pre- and post-operative management.

## MATERIAL AND METHODS

We studied all cases with primary VUR operated at our Unit of Ambulatory Surgery by ET with Deflux (dextranomer hyaluronic acid), from 2004 to 2009. The patients were diagnosed by at least one voiding cystouretrography and underwent at least two preoperative ultrasounds, to evaluate the rest of the urinary tract. All patients were studied for at least six months following the ET.

A total of 38 children were treated and their data on age, sex, reflux grade, laterality, and ET results in different degrees of reflux, was analysed. This was done in the case of children who had undergone endoscopic injection alone or those who had received two shots to reach the result of an ureter without reflux, as well. The post-operative complications were also studied.

All patients were diagnosed with having had at least one confirmed UTI, by implementing a conventional voiding cystouretrography. After diagnosis a urinary chemoprophylaxis protocol, approved by the Committee on Clinical Pharmacology of our hospital, was established, with cotrimoxazole or a cephalosporin in a prophylactic dose per day, in alternating cycles of 15 days. The prophylaxis was maintained until resolution of the VUR.

All children were evaluated at the pre-anaesthesia consultation, at the Paediatric Ambulatory Unit, on the morning of their treatment. The indications for surgery were recurrent UTIs or renal functional decline, registered in at least one renal scintigraphy.

**Figure 1:**
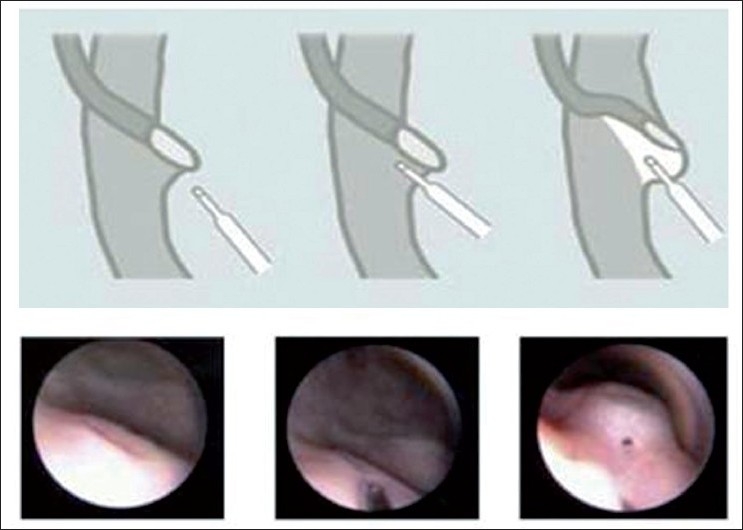
Deflux Injection, applied in ureteral submucosa at the lower edge of the ureteral meatus causing protuberance, remaining after applying in a fashion similar to a volcano

The endoscopic procedure was performed under general anaesthesia, using agent Propofol as the main anaesthetic, after intravenous administration of a cephalosporin prophylactic through a cystoscope, 9.5 F compact size, with a straight working channel, and a video to record the images and review them later. Deflux injection was applied in the sub-mucosa and the lower edge of the mouth intra-ureteral (in cases with high grade reflux) or ureteral, causing the protuberance of the ureteral orifice to the top of a mound caused by the correct application of dextranomer to that level, remaining after applying a similar fashion to a volcano [Figure 1]. The injection was given through a 5F metal needle, which was introduced into the straight channel of the cystoscope.

All the children were operated with conventional paediatric monitoring and were discharged on the day of surgery, while maintaining their analgesic treatment and urinary prophylaxis until the confirmation of the disappearance of reflux in a urethrocystography performed three months after treatment. Also, an abdominal ultrasound was performed in all cases at two post-operative weeks (which was repeated at four weeks if the first had some alteration) and at the sixth post-operative month. In cases where the first injection did not solve the problem, the patients were offered the possibility of a second outpatient treatment after the first six months.

## RESULTS

Over a period of six years, a total of 38 patients (25 girls (65%) and 13 children (35%)), were operated in our unit, aged between one and twelve years, with an average age of four years, and the largest age groups of two and three years, who made a total of 22 cases. VUR was bilateral in 24 (63%) patients and unilateral in 14 (37%) (eight left and six right), collecting a total of 62 renal units or ureters treated endoscopically.

Most patients had VUR grade IV, according to the international classification of VUR[3] with a total of 29 ureters (46%), VUR grade V was present in 17 ureters (25%) and grade III in 12 ureters (19%). The ureters with grade I (two cases, 5%) and II (two cases, 5%) were treated endoscopically, because they belonged to patients with bilateral VUR and with a contralateral ureter, which had a higher grade (IV – V) VUR.

Of the total of 62 ureteral units, in 29 children (76%) with 46 ureters (74%), reflux completely disappeared after only one ET [Table 1]. Nine patients (24%) with 16 ureteral units (30%) received a second ET, after which the reflux disappeared successfully in seven children (12 ureteral units), bringing the success rate in the disappearance of VUR, after two injections of Deflux, to 90% of the total group of ureters (58 of 62). The two patients (VUR grade V) without response after two endoscopic treatments were operated upon successfully later by an open antireflux surgery.

**Table 1:** Results after first Endoscopic treatment, Grade Ureters Success

I	2	2 (100%)
II	2	2 (100%)
III	12	9 (75%)
IV	29	25 (86%)
V	17	8 (47%)
Total	62	46 (74%)

Three children had complete renoureteral duplication. One of them being bilateral, we therefore had four double ureteral units receiving antireflux ET. In the two unilateral patients, the reflux was resolved with one injection and in a boy with bilateral duplication, after the second ET.

Post-operative follow-up was for a minimum of six months, and revealed a moderate dilatation of the renal pelvis on an ultrasound performed at two weeks of treatment, in five children (13%), which disappeared in the next control after four post-operative weeks. Post-operative complications included low back pain in the first week (two cases) and UTIs in three children in the first six months after ET.

The average time used for the surgical procedure was 40 minutes, and the average amount of Deflux used in each ureteral unit was 1.1 ml, with a volume range between 0.6 ml and 2.1 ml. The correlation between the volume of Deflux injected and the successful resolution of reflux was not found.

## DISCUSSION

The number of patients studied in this research, with VUR, showed a cure rate of 78% after one ET and up to 90% after a second endoscopic treatment. These results are very acceptable, as they coincide with those from other publications and are very close to the success rate achieved with open surgical treatment.[4,6–8] Furthermore, this technique can mean a surgical procedure, much less invasive, with an almost immediate postoperative recovery, as evidenced by our results and those of other recently published series and with a much lower postoperative complication rate.[8–10]

Likewise, another significant difference of this technique from open surgical procedures, is that it can be performed in a Paediatric Ambulatory Surgery Unit, with a consequent influence on the economic costs of the procedure, avoiding placement in a hospital bed to face the hard choice of surgery on a child and especially with greater acceptance and comfort of the patients and their families, as it decreases the separation time of the child from his parents, because it has been described in our group of patients and in most publications that the operative time is less than an hour.[7,8]

Not only are the child and family more comfortable, but the child in these units is not exposed to nosocomial organisms and their subsequent nosocomial infections.[11,12] Recent advances in paediatric anaesthesiology have also facilitated this process and many others in the field of paediatric urology, which can now be performed at ambulatory bases, well equipped for monitoring and administering drugs to children.[12,13] Moreover, another advantage of ET in the ambulatory setting, is that it can be included in the service portfolio of units that have pre-anaesthesia consultation and a clinical laboratory, capable of carrying out its assessments on the same day of admission and surgical treatment, being termed as a, ‘High Resolution Ambulatory Unit’. Some of them have been recently created in our country.[14]

Paediatric ambulatory surgery, took more than 65% of the surgical procedures for children in most Western countries,[12] with better results than those obtained when the same pathology was entered through hospitalisation. Therefore, it is essential to make a proper selection of patients for this type of organisation to achieve maximum quality results. Children must meet the physical criteria of the American Society of Anaesthesiology, ASA I and ASA II, to be anaesthetised with maximum safety in the operating room of the Ambulatory Surgery Unit.[13] Moreover, the type of patients we have described in this study should be carrying a primary VUR, as they were in our series of patients, questioning this treatment in situations of neurogenic bladder with secondary reflux or other malformations that may be associated with VUR, although there are some publications that also suggest its application in this population.[9]

Another essential point with these patients, are the instructions to parents or relatives at the time of discharge from the unit, which should be clear, to avoid confusion and unnecessary re-admissions. The children were discharged from the unit when they were in an appropriate state of vigilance, without nausea and with good pain control. It was hoped to undertake urination in the hospital, but it was not essential, because this could sometimes takeabout eight to ten hours, almost always depending on the appropriate dose of painkillers. According to some publications, the patient was not expected to drink liquids, as this depended also on the preferences of each child and each family, provided they were not discharged with nausea or vomiting.[12,14]

None of our patients attended the Emergency within 48 hours after surgery, although it has been described that there was re-entry of between 0.1 and 5% of the cases in some studies, which evaluated Paediatric Surgery Ambulatory Units.[12] The leading causes of re-entry, were usually urinary bleeding, vomiting and uncontrolled pain, and thus, parents had to monitor these symptoms, clearly and inform them to home care, as the presence of warning signs that should have account, before his appearance. Perhaps, one of the causes of the low incidence of vomiting in our study group was the use of propofol as the anaesthetic agent, as described elsewhere.[13]

Among the limitations that may be suggested in this study, and those that describe an experience with not a very large set of patients like ours, are, that the procedure depends on a learning curve, which can directly influence the outcome of the former as compared to the second period of treatment performed or when these patients have been operated by various surgeons.[4,5,9] This bias in the results, has been described in most published series, and is partially resolved when evaluating a number of patients treated in a significantly protracted time period, over five years, as has been in our case.

The endoscopic resolution of VUR has become the treatment of choice for the control of primary VUR in children, not only for its good results, but because of the low postoperative morbidity and a direct dependence on the Ambulatory Surgery Unit, which can provide proper pre- and post-operative management of children and their families. In the era of minimally invasive surgery, ET is a good example of innovation and development, compared to traditional open procedures that have been so successful in the recent history of Paediatric Urology. This innovative procedure can be successful in ambulatory bases, with the support of specialties related to the proper management of children, such as anaesthesia, paediatrics and nursing.
